# Synergistic Cytotoxic Stress and DNA Damage in Clover (*Trifolium repens*) Exposed to Heavy Metal Soil from Automobile Refining Shops in Kashmir-Himalaya

**DOI:** 10.5402/2011/109092

**Published:** 2012-01-05

**Authors:** Towseef Mohsin Bhat, M. Y. K. Ansari, Sana Choudhary, Rumana Aslam, Alka

**Affiliations:** Department of Botany, Faculty of Life Sciences, Aligarh Muslim University, Aligarh 202002, India

## Abstract

Coexposure to heavy metals occurs in many occupational settings, such as automobile refining shops, pigment, and batteries production. Heavy metals around automobile refining shops were tested for their ability to induce synergistic cytogenetic effects in *Trifolium repens* L. by using the chromosomal aberrasions (CAs), micronucleus (MN) and comet assay. A significant increase in micronucleus (MN), chromosomal abrations (CAs), percentage of nuclei with comet tails (NCTs), the relative comet tail length (CTL), comet tail DNA (CT, DNA), and tail moment (TM) were observed with increased concentration of three heavy metals, like Cd, Pb, Hg. The present result indicate that exposure of *T. repens* to soils contaminated by heavy metals around automobile refining shops shows clastogenicity, cytotoxicity, and DNA damage at higher concentrations.

## 1. Introduction

Plants experience oxidative stress upon exposure to heavy metals that leads to cellular damage. It is a well-known fact that many heavy metals are not mutagenic by themselves but converted by various metabolic pathways present in biological systems to derivatives that are reactive with DNA. Heavy metals are very much toxic to all living organisms, if they exceed human health limits [1]. Contaminated soil is a great threat for hyperaccumulated plants, which can affect human health [2, 3]. The soils of urban areas were mainly contaminated by the deposition of heavy metals which are emitted from industries, vehicles, and burning of wastes. Human activities like vehicular exhaust, lubricating oil residues, tyre wear particles, weathered street surface, and paint components contaminate the soil [4, 5]. The natural variability in soil composition is very well indicated by the presence of trace elements [6]. Certain heavy metals remain in the environment for an extensive period and eventually accumulate to levels that could harm physiochemical properties of soils and lead to loss of soil fertility and crop yield. Plants are good bioindicators of heavy metals because they play a significant role in food chain transfer and in defining habit. Furthermore, plant-based assays applied for toxicity evaluation in the field would reduce animal sacrifice and testing costs. The use of plant as bio-indicator for evaluation of genotoxicity has been reported in several studies [7, 8]. The heavy metal accumulation in the soil, however, has become a worldwide problem leading to reduced root and shoots growth, low-yield production, low-nutrient uptake, and impaired homeostasis. Growth inhibition is a general phenomenon associated with most of heavy metals [9, 10], while the tolerance limits for heavy metal toxicity are specific not only for species but also for each variety of crop plants [11, 12]. The heavy metals are frequently accumulated by agriculture important crops with a significant potential to impair animal and human health [13].

Although some studies have been done on the effect of heavy metals on plants, there is a very little information available on the effects of heavy metals on medicinal plants and grasses from Kashmir as Kashmir Himalaya is unique in its medicinal wealth and many medicinal varieties are becoming endangered due to their overexploitation, habitat destruction, and soil pollution. *T. repens* (2*n* = 32) a member of the fabaceae family is a perennial that is almost cultivated as a medicinal herb as well as fodder for live stock. *Trifolium repens* has been used in Indian ayurveda to treat number of diseases including Bright's disease [14]. It is a native of Europe and Asia, but has also been introduced to North America and New Zealand. In India it is grown as a pasture crop and is commonly known as white clover.

DNA is nature's most widely used long-term information storage system. The elegant simplicity of the double-helix belies the complex pathways that have evolved to copy, modify, and maintain the integrity of the genome. 

There are many common types of DNA damage that impact accurate replication by DNA polymerases [15]. Furthermore, the degree and spectrum of DNA damage depends on the sample source and the type of environment to which it is exposed. 

Comet assay (single cell gel-electrophoresis) is a simple and more reliable method for measuring DNA strand breaks in eukaryotes. Cells embedded in agarose on a microscope slide are lysed with detergent and high salt to form nucleotides containing supercoiled loops of DNA linked to the nuclear matrix. Electrophoresis at high pH results in structures resembling comets, observed by fluorescence microscopy; the intensity of the comet tail relative to the head reflects the number of DNA breaks. The assay has applications in testing novel chemicals for genotoxicity, monitoring environmental contamination with genotoxins, human biomonitoring and molecular epidemiology, and fundamental research in DNA damage and repair.

Therefore, present work was carried out in order to observe the effects of heavy metals from automobile refining shops on the medicinal cum fodder herb *T. repens*. In this study, our objectives were to determine the concentration of heavy metals in soil around the automobile refinishing shops and their cytotoxic effects on *Trifolium repens*. In this context, it becomes important to study the effect of heavy metals on medicinal plants particularly in which whole plant is used as a medicament. Therefore, *Trifolium *which has long history of uses in India as a medicine cum forage herb forms the subject matter of the present study.

## 2. Material and Methods

### 2.1. Sample Collection

The territory of the State of Jammu and Kashmir lies between four degrees of latitude from 32.17 to 36.58 at the North of India. Within these 640 kms, there is a sudden rise of altitude from 305 meters to 6910 meters above sea level. Generally in Kashmir the automobiles are repaired under trees in shade. We have collected 4 soil samples from different areas of Kashmir, India, near automobile refining shops from a distance of 1.5 km, and 1 soil sample for control was collected from the apple garden from a different area where there is no autobody refining shop.

### 2.2. Method

The surface soil (about 0–3 cm in depth) samples from each site and control locations were collected by the Teflon shallow scoop. The samples were stored in brown polyethylene bags, to avoid interaction of UV radiations.

### 2.3. Sample Preparation and Analysis

Samples were oven dried at 60 ± 2°C until moisture contents were removed. 5.0 gm of soil samples (2 mm sieved) were weighted and digested by wet oxidation method, using HNO_3_ (63%) at 61 ± 2°C.

After keeping at room temperature, the extract was filtered, and the filtrate was collected in a volumetric flask and brought to volume by distilled water. The concentrations of trace metals were estimated by Flame Atomic Absorption Spectrophotometer (A Analyst-700, PerkinElmer). Statistical analysis of data was also carried out.

### 2.4. Plant Growth Conditions

**Figure 1:**
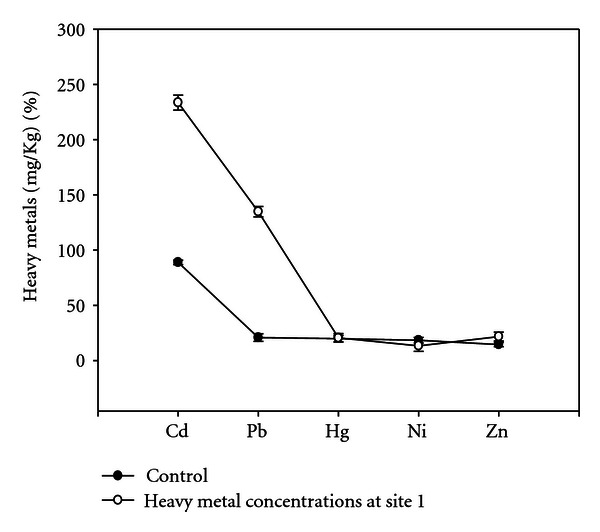
Concentrations of heavy metals at site 1.

**Figure 2:**
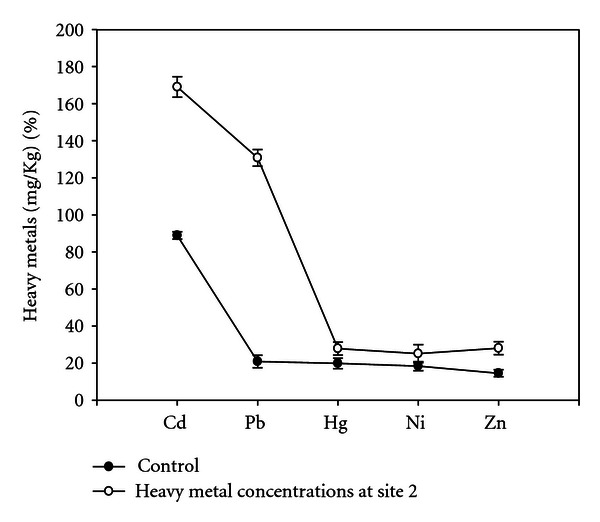
Concentrations of heavy metals at site 2.

**Figure 3:**
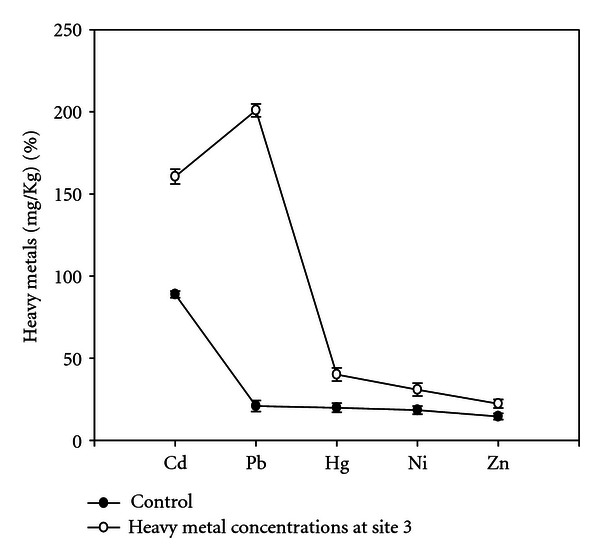
Concentrations of heavy metals at site 3.

**Figure 4:**
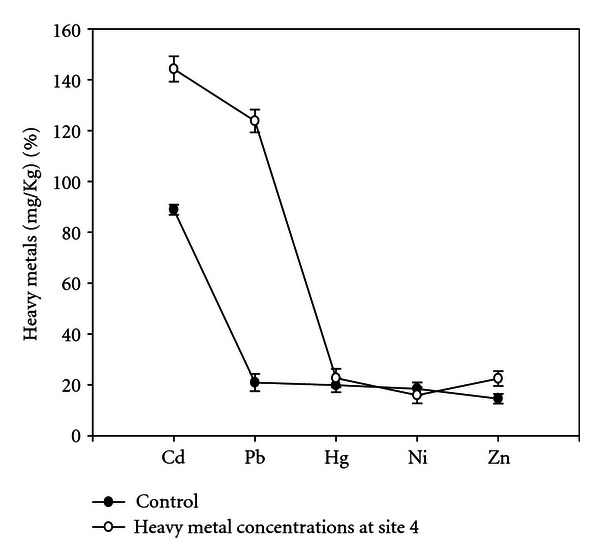
Concentrations of heavy metals at site 4.

The soil samples collected from different experimental sites when analyzed were having average pH 7.9, total nitrogen (%) 0.08, total phosphorus (%) 0.69, organic carbon (%) 1.981, and heavy metals (Figures 1, 2, 3, and 4). Four earthen pots (50 cm) were filled with 2 kg soil brought from different sites (S1 to S4) near automobile refining shops, and control pot was added 2 kg soil brought from apple garden. Three replicates for each concentration were used. Five seeds of same size were planted in each pot. The plants were watered daily and grown for a month, and care was taken for leaching of water by keeping plastic tub under each pot to collect the leachates. The collected leachates were again poured into the experimental pots. The plants were maintained at University net house under suitable light and temperature conditions. No supplements were added in collected soil at the time of plant growth.

### 2.5. Meiotic Analysis

Cytological studies of all the morphological variants of plants were carried out to study the effect of contaminated soil from automobile refining shops at chromosomal level. For cytological analysis, young flower buds (34 days old) from control as well as from contaminated soil were selected and fixed in Carnoy's fluid (alcohol : chloroform : acetic acid 6 : 3 : 1 ratio) for 40 minutes, transferred in propionic acid saturated with ferric acetate for 24 hrs, and then stored in 70% alcohol. Anthers were squashed in 0.5% propionocarmine. Slides were made permanent in NBA series, mounted in the Canada balsam, and dried at 45°C.

### 2.6. Micronucleus (MN) Test 

For MN analysis 400 cells were scored for each slide to calculate MN frequency. Micronucleated cells were evaluated under binocular light microscope (Olympus Japan). For scoring of MN, the method given by [16] was adopted.

### 2.7. Isolation of Plant Cell Nuclei

The protocols were provided by India Agriculture Research Institute, New Delhi, India. All experimental procedure was carried out in dim light to avoid DNA damage by light. After exposure to the contaminated soil, leaves to be processed were placed in deep freezer to keep them turgid, and then they were placed in a Petri dish on ice and spread with 250 *μ*L of cold 1×PBS (NaCl 130 mmol/L, Na_2_HPO_4_ 7 mmol/L, pH 7.0). By the help of a fresh blade, each leaf was cut into pieces. The pieces were washed into buffer by repeated pipetting using a micropipette. The plate was kept tilted in the ice, and the buffer was mixed so that the isolated nuclei would be collected in the buffer.

### 2.8. Comet Assay

The nuclei suspension was used in the alkaline single-cell gel electrophoresis assay, as described by Tomas and Matilde. The microscopic slides were firstly covered by 12 *μ*L of 0.4% normal melting point (NMP) agarose in phosphate buffer saline (PBS) kept at 65°C and were placed on the ice for 8 minutes to be solidified. This layer is necessary to secure a good adhesion to the slides of the mixture of LMA and nuclei. After the NMP solidification, the cover slips were gently moved. Then, 50 *μ*L of 1.0% low melting point (LMP) was added to 50 *μ*L of nuclei suspension by repeated pipetting. The mixture was immediately placed onto the slides with NMP. The corresponding large cover slips were immediately placed on the top of the cell gel mixture, and the slides were placed on ice for 7 minutes to allow solidification of agarose. The cover slips were gently removed, and 100 *μ*L of NMP were placed over the second agarose layer as the third layer. A new coverslip was placed on top and laced on a surface over ice for at least 10 minutes.

After the cover slips were removed, the slides were placed in a horizontal electrophoretic apparatus filled by cold alkaline buffer (265 mmol/L NaOH, 1 mmol/L Na_2_EDTA, pH = 16 at 4°C). The slides were immersed in the electrophoresis buffer to the depth of about 3.0 mm from the upper agarose surface for 30 minutes to allow the DNA to denature. Then electrophoresis was conducted at 4°C for 15 minutes at 300 Ma. After electrophoresis, the slides were neutralized with a neutralization buffer (0.4 mol·L^−1^ tris HCl, pH 7.5) at room temperature for 13 minutes. Then the slides were immersed in ethanol at room temperature for 1 h and put in the dark to be air dried.

The following parameters were used as primary agent for DNA damage: percentage of nuclei with tails, the relative tail length, tail DNA (relative percentage of DNA in the comet tail), tail moment (TM, integrated value of density multiplied by migration distance).

## 3. Results

### 3.1. Meiosis

**Figure 5:**
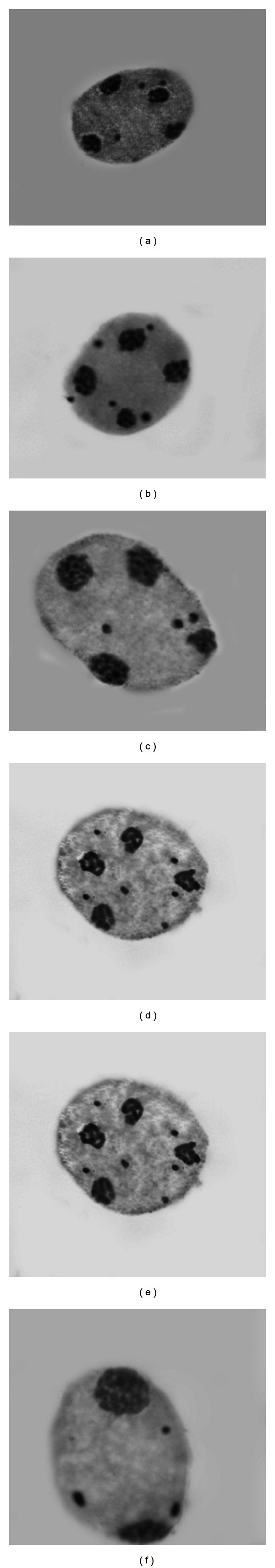
(a)–(e) Micronucleus condition. (f) Precocious separation and stickiness at metaphase I.

Heavy metal concentrations from different experimental sites were given in (Figures 1–4), the heavy metals which are present in higher quantity were cadmium, lead, and mercury, and heavy metals like nickel and zinc were present in trace amounts; therefore, their effect was considered as negligible. Meiosis was perfectly normal in the control plants. However, the plants grown in soils of different sites develop varying degrees of chromosomal abnormalities disturbed in all phases of division. A dose-based increase in meiotic abnormalities distributed in all phases of division with respect to increased heavy metal concentration of different sites (Figures 5(a)–5(f)).

The most prominent abnormality induced by heavy metals were micronucleus, precocious separation, and laggards.

### 3.2. Effect of Heavy Metals on MN Frequency

**Figure 6:**
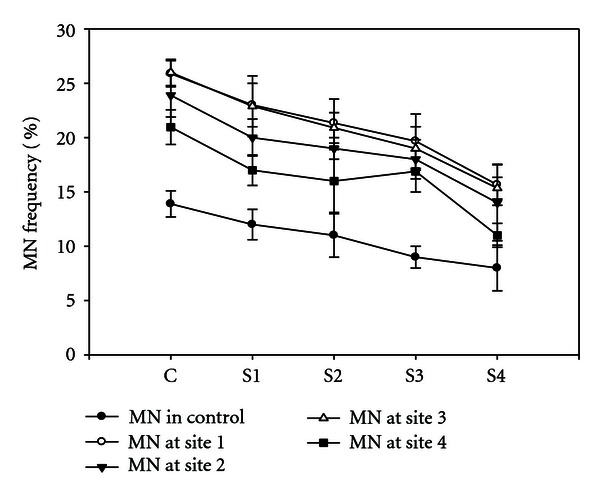
MN frequency observed in *V. odorata* by heavy metals from different sites with respect to control.

The MN frequency results showed that heavy metals induce MN frequency in all the plants grown from different sites except in the control. The increasement of MN frequency from different sites was concentration dependent. The highest frequency was observed from the site having highest heavy metal concentration (Figure 6).

### 3.3. Percentage of Nuclei with Tail

**Figure 7:**
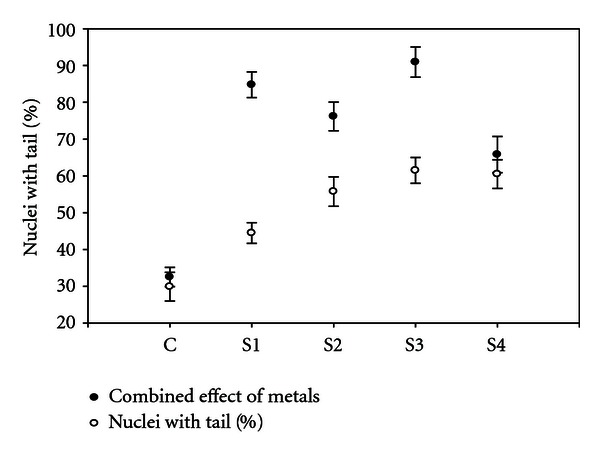
Percentage of nuclei with tail by the effect of heavy metals from different sites and control. Error bars are SE.

The percentage of nuclei with tails increased with the increasement in concentration of heavy metal (Figure 7). The highest percentage of nuclei with tails was associated in decreasing order (S3 > S1 > S2 > S4 > C).

### 3.4. DNA Migration after Exposure to Heavy Metals

**Figure 8:**
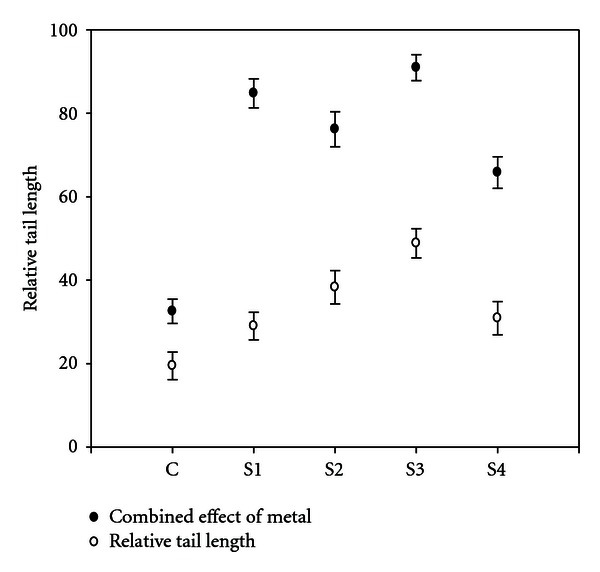
Relative tail length of DNA by heavy metals from different sites and control. Error bars are SE.

After the alkaline comet assay, the DNA migration was expressed by relative tail length, tail DNA, tail moment, as a rough estimation of DNA migration, and the length of the comet tails shown in Figure 8. It was seen that, with increase in heavy metal concentration, the mean comet tail length increases showing a clear concentration relationship.

**Figure 9:**
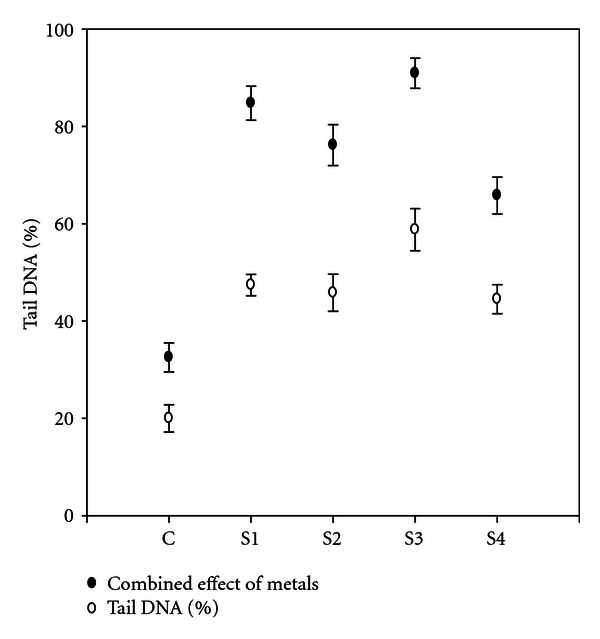
Percentage of nuclei with tails by heavy metals from different sites. Error bars are SE.

Tail DNA (relative % of DNA in the comet tail) appeared to be the most informative parameter of DNA damage. It was found that tail DNA increased with the increasing heavy metal concentration (Figure 9) and was found to be highest in site 3 (S3) followed by site 1 (S1), site 2 (S2), and site 4 (S4).

**Figure 10:**
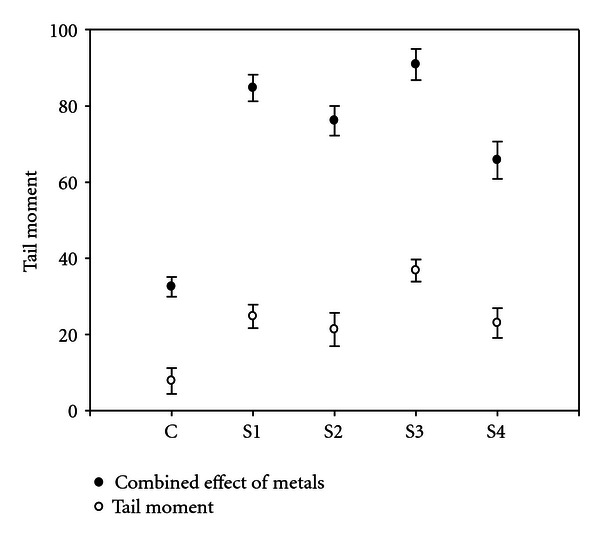
Tail moment of DNA due to heavy metals from different sites and control. Error bars are SE.

Tail moment (TM) increases with increasement of heavy metal concentration. Site 3 (S3) has the highest concentrations of heavy metals followed site 1 (S1), site 4 (S4), and site 2 (S2) (Figure 10).

## 4. Discussion

Assessment of the ecological and genetic impact of heavy metal pollution on plant populations is of great importance as plants provide important commercial products and are used by people as well as by the livestock. Moreover, plants may be used as biosensors of genetic toxicity of environmental pollutants. In this research by meiotic analysis, MN frequency, and comet assay, the potential for DNA damage in *T. repens* L. as a biomarker of genetic toxicity was considered. The sensitivity of the single-cell gel electrophoresis (SCGE) or comet assay allows rapid prediction of genotoxic potential of compounds and has been shown to be useful for in vivo and in vitro biomonitoring of environmental pollutants [17, 18]. In the present study, the *T. repens* plants grown on the contaminated soil of automobile refinishing shops having heavy metal concentration showed assessments of DNA damage, since the beginning of the industrial revolution [19].

In the present study, the methodology involves breaking the cell wall mechanically and isolation of the nucleus. The *Trifolium repens* leaf cell nuclei were found to be more convenient and authentic than other methods because in this method the plant accumulate more metabolites in its leaves, as *T. repens* is a medicinal plant and the essential parts being the flowers and leaves from which different chemicals work against the various ailments. The operation such as the preparation of microscopic slides was more authentic and throws light on DNA damage cytologically [20, 21].

In this present study, the effects of heavy metals from automobile refining shops and compared with control were obvious, and it was seen that there is a positive relationship between increased heavy metal concentration and DNA breakage which reflects in the formation of micronucleus, laggards, and comets. The present study also throws light that, if heavy metals from autobody refining shops, will remain unchecked can accumulate in the food chain, as *Trifolium repens* is grown as a medicinal and pasture plant consumed by the live stock, on which humans depend for milk, meat, and so forth [20, 22].

In the present study, although all the five sites vary in their heavy metal concentrations, it may be because of efficiency of automobile repairers. Currently, environmental risk assessment of contaminated soils is based on analytical methods which reveal the presence of many mutagenic and carcinogenic compounds in the soil like heavy metals, PAHs, pesticides, and so forth. A major limitation of this approach is that most of the compounds present in soil are still unknown, and most soil ecotoxicological data relates to relatively less known compounds [23]. This inadequacy stipulates the inclusion of different bioassays for evaluation of genotoxic potential of soils as biomonitors in health hazards assessment. Many studies of genotoxicity and mutagenicity of different soil samples have been conducted employing the Ames test [24, 25], *Tradescantia *micronucleus test [8, 26], *Tradescantia* stamen hair mutation assay [27, 28]; among these, the most authentic test we found was comet assay which provide appropriate results.

Heavy metals may enter into the cell nucleus and may bind to purine and pyrimidine bases or proteins, denature spindle, and cause MN formation as a result of decrease in the chromosome number in the main nucleus [29].

An important question about heavy metals in the environmental concerns is the amount that plants can tolerate and accumulate without adverse effects. A further question relates more to the amount and speciation and their roles for plant performance and metal transfer to the food chain. 

## 5. Conclusion

Many occupations and professions which are running in industrial and residential areas pose great threat to the biodiversity. The “autobody refinishing” is such kind of occupation which largely affects the environment; particularly the painting and scrapping processes were done without any safety measures. Metal pigments in paints and scrap are continuously deposited with effects on the workers and on soil. The results of the present study showed that the soil around these autobody refinishing areas were contaminated exponentially due to gradually increase in the autobody refinishing shops which is a great threat to the environment. The levels of Cd, Pb, Hg, Mn, Fe, Cr, Zn, Co, Ni, Mo, and Cu contents were higher than the control samples. It shows an alarming situation of their increase in soil. The remedial measures will be adopted to reduce the level of metal contents, by scientific observations. Glutathione is a well-known player which works against heavy metals by detoxifying ROS (reactive oxygen species) through ascorbic acid-glutathione cycle. The accumulated metals are detoxified by phytochelatins, which are synthesized from glutathione in plants when they are exposed to heavy metals. Phytochelatins form complex with heavy metals and are sequestered into vacuole. Efforts should be taken up to find out the functional validation of repair proteins through transgenic approach. Overall, a better understanding of DNA damage response and the mechanism of DNA repair in plants should contribute to the solution of impeding environmental and social problems.
